# Timing of retinal neuronal and axonal loss in MS: a longitudinal OCT study

**DOI:** 10.1007/s00415-016-8127-y

**Published:** 2016-05-03

**Authors:** Lisanne J. Balk, Andrés Cruz-Herranz, Philipp Albrecht, Sam Arnow, Jeffrey M. Gelfand, Prejaas Tewarie, Joep Killestein, Bernard M. J. Uitdehaag, Axel Petzold, Ari J. Green

**Affiliations:** Department of Neurology, MS Centre, VU University Medical Center, Mailbox 7057, 1007 MB Amsterdam, The Netherlands; Department of Neurology, University of California San Francisco, San Francisco, USA; Department of Neurology, Heinrich-Heine University, Düsseldorf, Germany; Moorfields Eye Hospital, City Road, London, UK; UCL Institute of Neurology, Queen Square, London, UK

**Keywords:** Multiple sclerosis, Optical coherence tomography, Neurodegeneration, Retina, RNFL

## Abstract

The objective of the study was to investigate the timing of central nervous system tissue atrophy in MS by evaluating longitudinal retinal volume changes in a broadly representative cohort with disease duration across the entire arc of disease. In this longitudinal study, 135 patients with MS and 16 healthy reference subjects underwent spectral-domain optical coherence tomography (OCT) at baseline and 2 years later. Following OCT quality control, automated segmentation of the peripapillary retinal nerve fiber layer (pRNFL), macular ganglion cell–inner plexiform layer (mGCIPL) and macular inner nuclear layer (mINL) was performed. Generalized estimation equations were used to analyze longitudinal changes and associations with disease duration and clinical measures. Participants had a median disease duration at baseline of 16.4 years (range 0.1–45.4). Nearly half (44 %) of the MS patients had previously experienced MS-related optic neuritis (MSON) more than 6 months prior. The MS patients demonstrated a significant decrease over 2 years of the pRNFL (−1.1 µm, 95 % CI 1.4–0.7, *p* < 0.001) and mGCIPL (−1.1 µm, 95 % CI −1.4 to −0.8, *p* < 0.001). This thinning was most pronounced early in the course of disease. These findings were irrespective of previous episodes of MSON. No consistent pattern of change was observed for the mINL (−0.03 µm, 95 % CI −0.2 to 0.2, *p* = 0.795). This longitudinal study demonstrated that injury of the innermost retinal layers is found in MS and that this damage occurs most rapidly during the early stages of disease. The attenuation of atrophy with longer disease duration is suggestive of a plateau effect. These findings emphasize the importance of early intervention to prevent such injury.

## Introduction

Neuroaxonal loss is thought to be the primary substrate underlying irreversible disability in multiple sclerosis (MS) [1, 2]. However, the timing of neuroaxonal loss in MS is unknown, with injury detected early [3, 4], but more severe atrophy seen at later time stages [5–7]. With renewed interest in developing treatments for progressive MS, and interest in using atrophy measures as a potential biomarker for clinical trials in progressive disease, it is crucially important that the timing of tissue loss in MS is established.

The anterior visual pathway provides a discrete anatomically segregated system to investigate the dynamics of neuroaxonal degeneration in MS [8, 9]. Injury to axons and neurons in the retina can be quantified by retinal spectral-domain optical coherence tomography (SD-OCT). In addition, given the very high degree of reproducibility of OCT, it is a potential method for tracking neuroaxonal loss in MS at the level of the individual patient. Cross-sectional studies using SD-OCT have demonstrated evidence of injury to the inner retinal layers in MS, such as the peripapillary retinal nerve fiber layer (pRNFL) and the macular ganglion cell–inner plexiform layer (mGCIPL) [10–14], and established that this injury is correlated with clinical disability [11, 14–16]. Although retinal injury is most severe in the later stages of the disease [17–20], retinal axonal loss has been identified at early stages in the disease course [21–24]. In a large-scale histology study, it was shown that retinal injury not only includes axonal loss (thinning of the pRNFL), but also a reduction in ganglion cell density, demonstrating that inner retinal thinning in MS reflects neuronal and axonal loss [20]. However, the timing and pace of this injury, as with the rest of the central nervous system, remains unknown. Therefore, the main objective of this study was to investigate longitudinal changes in pRNFL, mGCIPL and macular inner nuclear layer (mINL) thickness over a 2-year period in a broadly representative cohort of patients with a wide range of disease duration in comparison with a healthy reference group. This study was designed to evaluate whether the rate of retinal layer thinning was dependent on disease duration and if the pace of decline was correlated with clinical disability.

## Methods

This study was approved by the Medical Ethical Committees on Human Research of the VU University Medical Center in Amsterdam, the Netherlands, and the University of California, San Francisco Committee on Human Research and is in accordance with the 1964 Declaration of Helsinki. Written informed consent was obtained from all subjects before study inclusion.

### Study design and patient population

In order to investigate the dynamics of retinal layer injury, a broadly representative cohort of patients with MS was included in this longitudinal multi-center observational study. Patients were enrolled from the VU University Medical Center Amsterdam (VUMC) and University of California, San Francisco (UCSF). Additionally, a sample of healthy reference subjects was enrolled at the VU University Medical Center Amsterdam for comparison.

All subjects were required to be between 18 and 80 years of age at the time of their baseline assessment. MS patients were included if they had ‘high-risk CIS’ (defined as a history of one clinical demyelinating relapse and at least one T2 hyperintensity typical of demyelination on conventional brain MRI), relapsing remitting MS (RRMS), secondary progressive MS (SPMS) or primary progressive MS (PPMS) at the time of their baseline assessment (as defined by Lublin–Reingold criteria) [25].

All subjects underwent clinical and OCT assessments at baseline and were retested after 24 months (with a 4-month visit window). Disease duration was defined as the time from the first MS symptom. The Expanded Disability Status Scale (EDSS) [26] was obtained by a certified examiner. As potential swelling of the pRNFL during the acute stages of MS related optic neuritis (MSON) may confound OCT measurements, patients were excluded if they had experienced symptomatic MSON 6 months prior to either OCT measurement (baseline or follow-up), according to a standard care protocol [27].

### SD-OCT

**Fig. 1:**
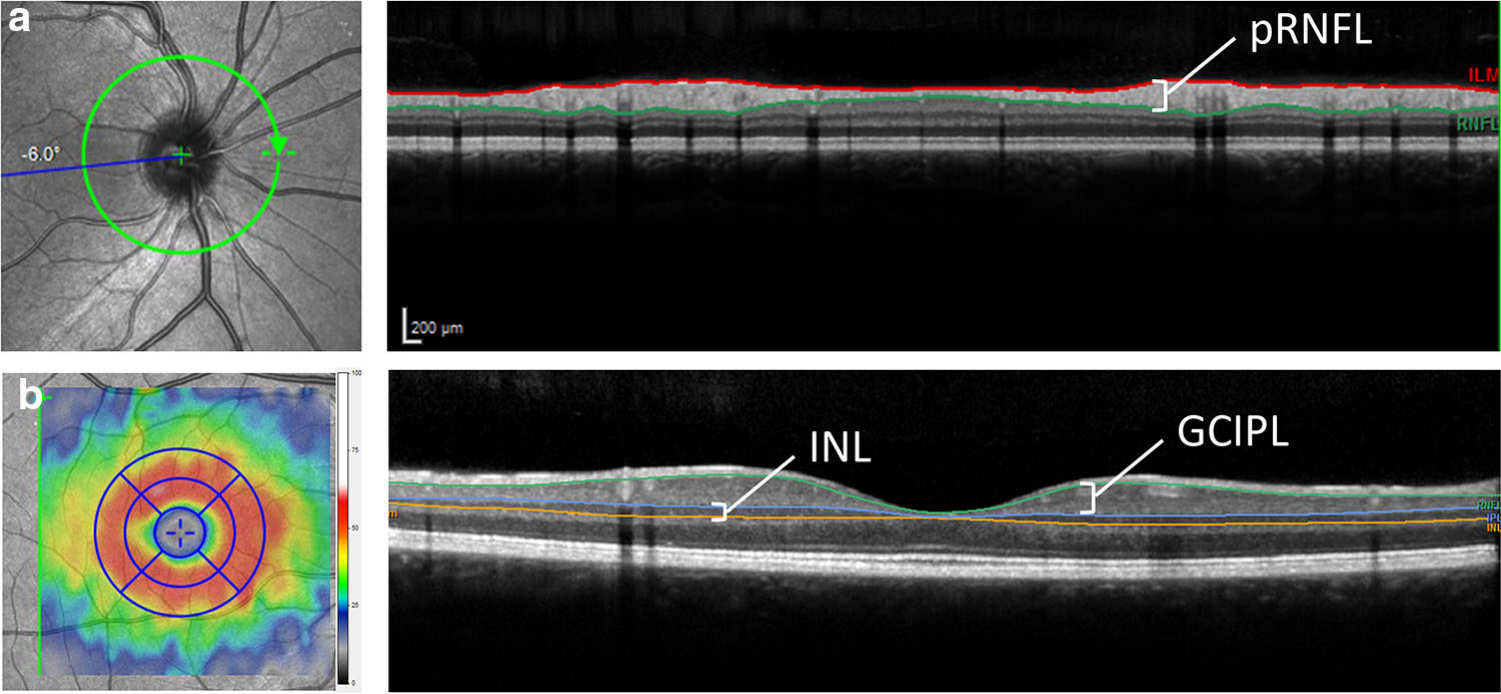
Scan and segmentation protocol for the OCT scans used in this study. **a** The peripapillary ring scan (12° diameter) was used to quantify the global mean retinal nerve fiber layer (pRNFL) thickness. **b** The macular volume scan (20° × 20° field, 25 B-scans) was centered on the fovea and used to assess thickness of the macular ganglion cell + inner plexiform layer (mGCIPL) and the inner nuclear (mINL). The colored map represents a change in thickness map with a 1, 2.22 and 3.45-mm EDTRS grid of which the eight perimacular sectors were used in this study. The inner 1 mm circle was excluded from the analysis because of absence of mGCIPL and mINL in the fovea

Spectral Domain OCT (SD-OCT, Spectralis, Heidelberg Engineering, Heidelberg, Germany) was performed in all subjects at baseline and follow-up. In all scans, eye tracking function was enabled for optimal image registration for averaging and follow-up [28]. For all follow-up scans, automatic scan placement was used with the baseline scan as reference. Data on the pRNFL were obtained using a 12° ring scan placed around the optic nerve head (Fig. 1A). Data on the macular area (mGCIPL and mINL) were acquired using a macular volume scan (20° × 20° field, 49 B-scans) centered on the fovea (Fig. 1b).

Scans were excluded from the analyses if they did not fulfill the revised quality control criteria (OSCAR-IB) [29]. In order to assess retinal pathology potentially resulting in exclusion of OCT scans, the medical history of visual symptoms was obtained from all patients and controls. In particular, the assessment of history of symptomatic ON was based on a standard protocol [27] and presence of microcystic macular edema (MME) was assessed according to diagnostic criteria described by Burggraaff et al. [30]. Following this OCT quality control, automated segmentation of the pRNFL, mGCIPL and mINL was performed (Heidelberg Engineering, software version 1.7.1.0). In addition, patients underwent clinical and ophthalmological evaluations for assessment of physical and visual function. Baseline Multiple Sclerosis severity scale (MSSS) scores were calculated for all patients, based on baseline EDSS scores and disease duration.

### Statistical analyses

Differences in disease duration and MSSS scores between the two participating centers were tested using the non-parametric Mann–Whitney *U* test and the independent sample *T* test, respectively. Longitudinal changes in retinal layer thickness were treated as absolute change scores (Δ, delta) and relative change scores (percentage change) over the 2-year observation period. Change scores were used to correct for biases caused by potential center-effects. All analyses performed on eye-level (two separate eyes per subject) were done using generalized estimation equations (GEE), with an exchangeable correlation matrix and adjustments for intra-subject inter-eye correlations. Additional adjustments for confounding factors such as age, sex and disease type were performed as indicated. In order to investigate the association with disease duration (defined by time since first symptoms), this variable was categorized into six groups based on the following cut-off values: <5 years (*N* = 31), 5.0–9.9 years (*N* = 15), 10.0–14.9 years (*N* = 15), 15.0–19.9 years (*N* = 30), 20.0–24.9 (*N* = 17) and ≥25 years (*N* = 26).

## Results

**Fig. 2:**
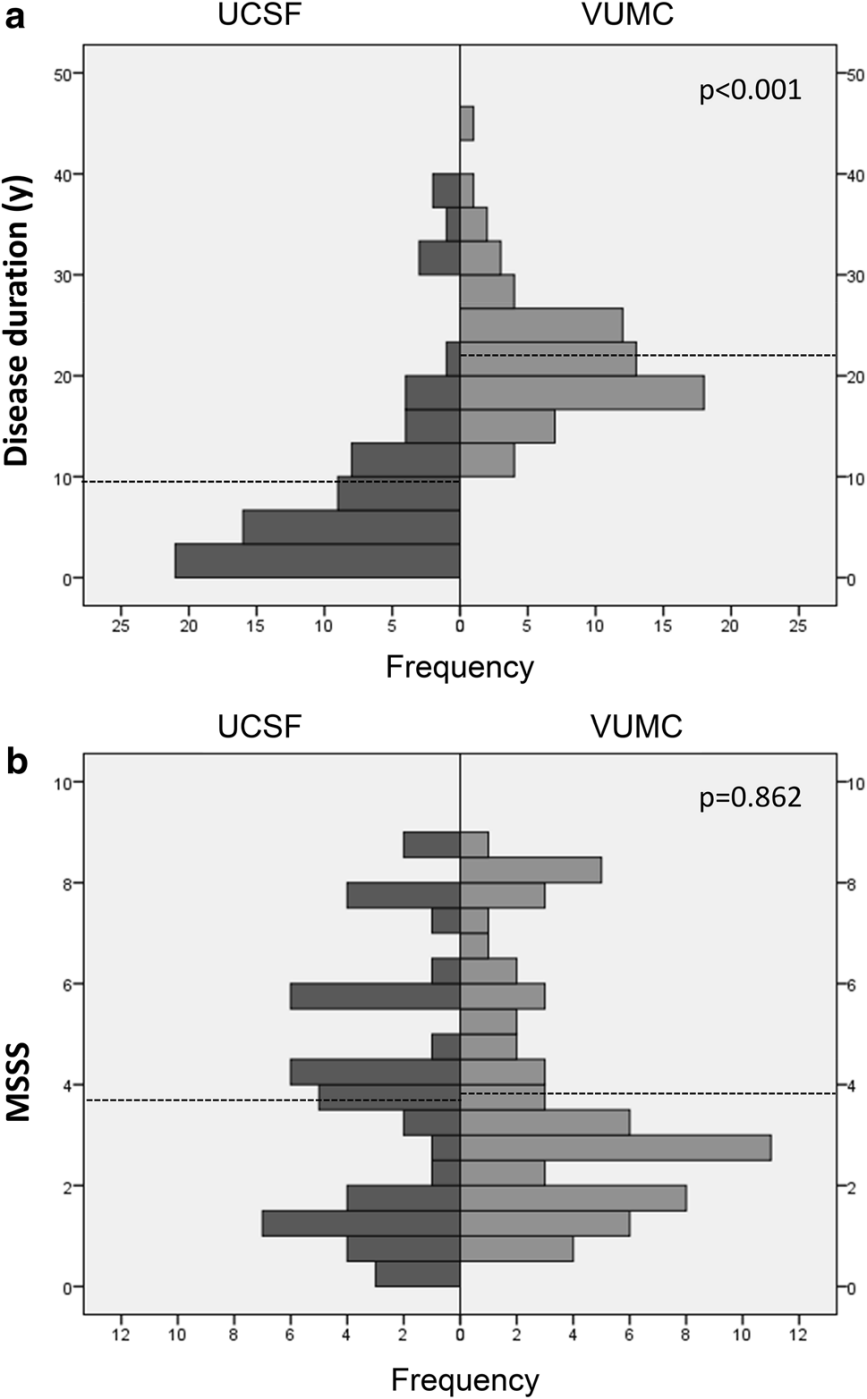
Center specific distribution of **a** disease duration (years) and **b** disease severity (MSSS score). Patients recruited from the VUMC Amsterdam had a significantly longer disease duration [22.0 years (SD 6.5)] compared with patients from UCSF [9.2 years (SD 9.6), *p* < 0.001]. Disease severity (MSSS score) was comparable for both centers [UCSF 3.7 (SD 2.5) and VUMC 3.8 (SD 2.4), *p* = 0.862]. The *horizontal dotted lines* indicate the mean value per center

A total of 135 MS patients (7 high-risk CIS, 89 RRMS, 26 SPMS, 13 PPMS) were included in this longitudinal study (*N* = 65 from VUMC and *N* = 70 from UCSF). At baseline, patients had a median disease duration of 16.4 years, with a range extending from 0.1 to 45.4 years. Although disease duration at baseline was shorter for patients enrolled at UCSF compared with VUMC (*p* < 0.01, Fig. 2a), disease severity (reflected by baseline MSSS scores) was similar for patients from UCSF and VUMC (*p* = 0.862, Fig. 2b).

Disease-modifying treatment was used by 58 patients (43.0 %). Nearly half of all patients had previously experienced a clinically identified episode of unilateral (30.4 %) or bilateral (most sequential) (13.3 %) MSON. Presence of MME was not identified in any of the patients, according to validated criteria [30]. Eight eyes were excluded because of an episode of ON in the 6 months prior to the OCT measurement. None of the included patients had experienced an episode of ON between baseline and follow-up measurement (more than 6 months prior to the follow-up visit). An overview of the clinical and demographic baseline data is shown in Table 1. Additionally, a sample (*N* = 16) of healthy reference subjects was analyzed. Of these 16 subjects, 7 (44 %) were female and they had a mean age of 50.7 years (SD 7.1). For all subjects, the mean follow-up period was 24.4 months (SD 1.8).

**Table 1:** Baseline characteristics

	MS patients *N* = 135
Sex (*N* [%] female)	101 (75.0 %)
Age (years, mean [SD])	49.2 (SD 12.1)
Disease duration (years, median [range], IQR)	16.4 [0.1–45.9], IQR 16.3
Disease type (*N* [%])	
High-risk CIS	7 (5.2 %)
RR	89 (65.9 %)
SP	26 (19.3 %)
PP	13 (9.6 %)
EDSS score (median [range], IQR)	3.0 [0.0–8.0], IQR 2.5
MSSS score (mean [SD])	3.75 (SD 2.4)
Disease-modifying treatment	
Current	58 (43.0 %)
Interferon/glatiramer acetate	42 (72.4 %)
Natalizumab	9 (15.5 %)
Other	7 (12.1 %)
Past	25 (18.5 %)
Never	40 (29.6 %)
Unknown	12 (8.9 %)
History of optic neuritis (*N* [%])	
None	72 (53.3 %)
Unilateral	41 (30.4 %)
Bilateral	18 (13.3 %)
Unknown	4 (3.0 %)
Time between baseline and follow-up (months, mean [SD])	24.4 (SD 1.8)
OCT measurements	
pRNFL (µm)	83.8 (SD 13.6)
mGCIPL (µm)	77.5 (SD 13.8)
mINL (µm)	38.9 (SD 3.6)

The number (percentage, %), median [range] and mean [standard deviation, SD] are shown

*CIS* clinically isolated syndrome, *RR* relapsing remitting, *SP* secondary progressive, *PP* primary progressive, *IQR* interquartile range, *EDSS* Expanded Disability Status Scale, *MSSS* multiple sclerosis severity score, *pRNFL* peripapillary retinal nerve fiber layer, *mGCIPL* macular ganglion cell + inner plexiform layer, *mINL* macular inner nuclear layer

### Longitudinal changes in retinal layer thickness

Table 2 shows the mean individualized changes in pRNFL, mGCIPL and mINL thicknesses over the 2-year observation period. Analyses with all MS eyes included, with a correction for intra-subject inter-eye correlations, demonstrated a significant decrease for both the pRNFL (−1.1 µm) and mGCIPL (−1.1 µm, *p* < 0.001 for both comparisons) over a 2-year period. No significant changes were observed for the mINL (−0.03 µm, *p* = 0.795). Importantly, the observed thinning in pRNFL and mGCIPL thickness in patients with MS was significantly larger compared with the healthy reference group (*p* = 0.015 and 0.018, respectively), which showed −0.5 µm thinning for the mGCIPL and −0.1 µm for pRNFL.

**Table 2:** Individualized retinal layer changes over time, stratified by MS patients and healthy controls, MS eyes with and without previous ON and relapsing MS and progressive MS

	Absolute change (with 95 % CI)	Relative change (with 95 % CI)	*p* value^b^
All MS eyes^a^			
pRNFL µm (*N* = 239 eyes)	**−1.1 (−1.4 to −0.7)**	**−1.3** **% (−1.7 to −0.9)**	**<0.001**
mGCIPL µm (*N* = 248 eyes)	**−1.1 (−1.4 to −0.8)**	**−1.3** **% (−1.7 to −0.9)**	**<0.001**
mINL µm (*N* = 248 eyes)	−0.03 (−0.2 to 0.2)	−0.03 % (−0.6 to 0.5)	0.795
Healthy references			
pRNFL (*N* = 31 eyes)	−0.1 (−0.8 to 0.6)	−0.1 % (−0.8 to 0.5)	0.797
mGCIPL (*N* = 30 eyes)	**−0.5 (−0.9 to −0.1)**	**−0.5** **% (−1.0 to 0.0)**	**0.021**
mINL (*N* = 30 eyes)	+0.4 (−0.1 to 0.9)	+1.0 % (0.0 to 2.2)	0.082
MSNON eyes			
pRNFL µm (*N* = 168 eyes)	**−1.0 (−1.4 to −0.5)**	**−1.1** **% (−1.5 to −0.6)**	**<0.001**
mGCIPL µm (*N* = 174 eyes)	**−0.9 (−1.2 to −0.6)**	**−1.1** **% (−1.5 to −0.7)**	**<0.001**
mINL µm (*N* = 174 eyes)	−0.04 (−0.3 to 0.2)	−0.03 % (−0.8 to 0.7)	0.769
MSON eyes			
pRNFL µm (*N* = 64 eyes)	**−1.4 (−2.0 to −0.7)**	**−1.7** **% (−2.5 to −1.0)**	**<0.001**
mGCIPL µm (*N* = 74 eyes)	**−1.2 (−2.0 to −0.5)**	**−1.7** **% (−2.8 to −0.7)**	**<0.001**
mINL µm (*N* = 74 eyes)	−0.06 (−0.5 to 0.3)	−0.1 % (−1.1 to 0.9)	0.862
RRMS eyes			
pRNFL (*N* = 170)	**−1.4 (−1.8 to −1.0)**	**−1.6** **% (−2.1 to −1.1)**	**<0.001**
mGCIPL (*N* = 176)	**−1.1 (−1.5 to −0.7)**	**−1.4** **% (−1.9 to −0.9)**	**<0.001**
mINL (*N* = 176)	−0.08 (−0.3 to 0.2)	−0.15 % (−0.8 to 0.5)	0.486
SPMS eyes			
pRNFL (*N* = 48)	−0.3 (−1.0 to 0.4)	−0.3 (−1.2 to 0.6)	0.395
mGCIPL (*N* = 48)	−0.8 (−1.7 to 0.01)	−1.2 (−2.4 to −0.0)	0.053
mINL (*N* = 48)	−0.1 (−0.5 to 0.3)	−0.3 (−1.3 to 0.8)	0.598
PPMS eyes			
pRNFL (*N* = 21)	+0.01 (−1.0 to 1.0)	+0.1 % (−1.1 to 1.2)	0.987
mGCIPL (*N* = 24)	−0.4 (−1.5 to 0.6)	−0.5 (−1.7 to 0.6)	0.448
mINL (*N* = 24)	0.8 (−0.3 to 1.8)	+2.0 (−0.7 to 4.7)	0.155

Bold values indicate *p* < 0.05

*pRNFL* peripapillary retinal nerve fiber layer, *mGCIPL* macular ganglion cell + inner plexiform layer, *mINL* macular inner nuclear layer, *ON* optic neuritis, *RRMS* relapsing remitting MS, *SPMS* secondary progressive MS, *PPMS* primary progressive MS

^a^Scans which did not fulfill quality control criteria (OSCAR-IB) were excluded from the analyses

^b^Unadjusted GEE analysis (only intercept models) using the absolute change scores

Subsequently, analyses stratified for MS eyes with (MSON) and without (MSNON) symptomatic ON showed that longitudinal changes in pRNFL, mGCIPL and mINL thickness were similar for MSON and MSNON eyes. The data demonstrate that previous episodes of MSON did not significantly affect the rate of thinning during the 2-year observation period for pRNFL (−1.0 µm MSON vs −1.4 µm MSNON, *p* = 0.125) mGCIPL (−0.9 µm MSON vs −1.2 µm MSNON, *p* = 0.454) or mINL (−0.04 µm MSON vs −0.06 µm MSNON, *p* = 0.895).

When the change scores for the 2-year observation period were stratified according to disease type, a significant difference emerged between relapsing MS and progressive MS (SPMS and PPMS combined) for the pRNFL (*p* = 0.002), but not for the mGCIPL (*p* = 0.191) and mINL (*p* = 0.540). In relapsing MS, significant thinning was observed for the pRNFL (−1.4 µm, 95 % CI −1.8 to −1.0), which was 14 times more than observed in the reference group (−0.1 µm). In SPMS the rate of atrophy over time for the pRNFL was significantly less (−0.3 µm, 95 % CI −1.0 to 0.4), but still three times larger than in healthy reference subjects. For the mGCIPL, the atrophy rate was slightly higher for the RRMS group (−1.1 µm, 95 % CI −1.5 to −0.7) compared to SPMS group [−0.8 µm (−1.7 to 0.01), *p* = 0.053]. Regarding the mINL, neither relapsing nor progressive MS patients showed significant consistent changes over the 2-year observation period across the cohort.

### Changes in retinal layer thickness are more pronounced early in the course of disease

**Fig. 3:**
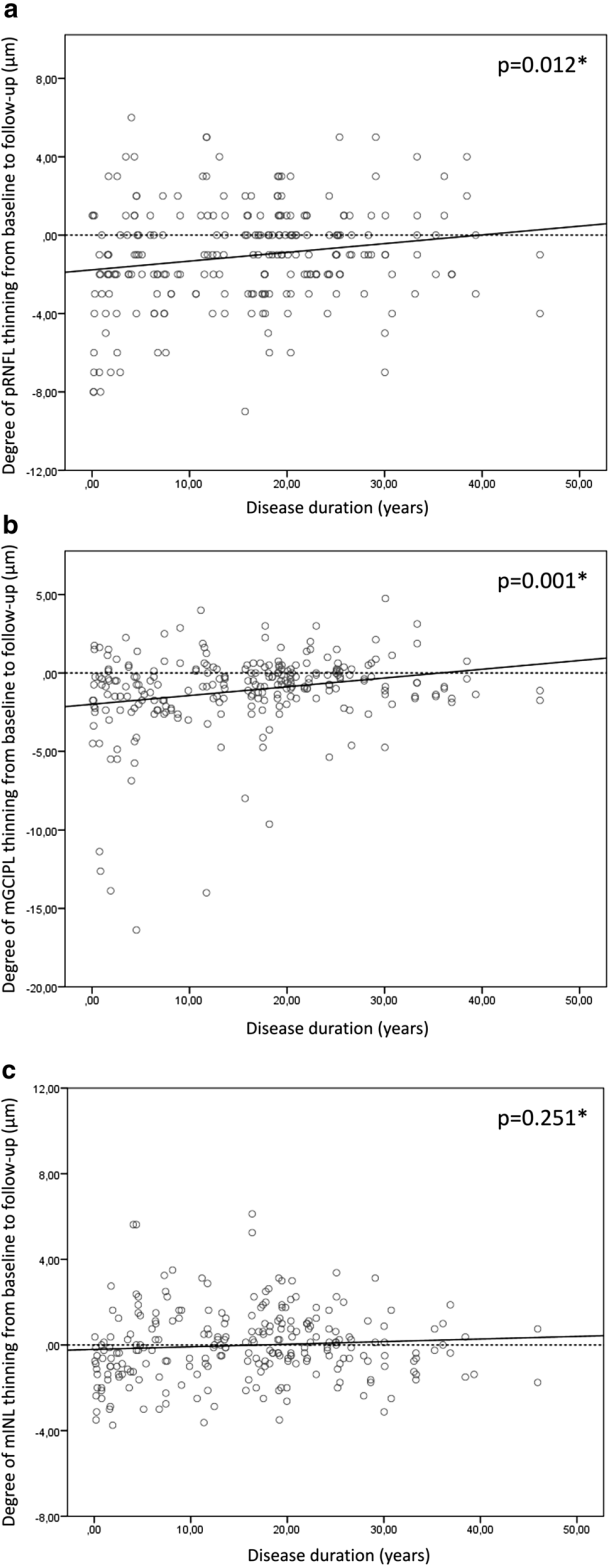
Association between disease duration and change in retinal layer thickness. Thinning of the pRNFL **a** and mGCIPL **b** is significantly associated with disease duration. For the mINL (C), no significant association was observed. * GEE, adjusted for inter-eye correlation, optic neuritis, sex

Thinning of the pRNFL and mGCIPL was significantly related to disease duration [*β* 0.05, 95 % CI 0.01–0.09, *p* = 0.012 and *β* 0.06, 95 % CI 0.03–0.10, *p* = 0.001, respectively (GEE, adjusted for inter-eye correlation, optic neuritis and sex)]. Because change scores were negative (which indicates thinning), the positive regression coefficients demonstrate that with a longer disease duration, the rate of thinning becomes smaller. More specifically, for every additional year of disease duration, the rate of thinning over a 2-year period, decreases by 0.05 µm for the pRNFL and 0.06 µm for the mGCIPL. Changes in mINL thickness did not show a relationship with disease duration. The associations between retinal layer thinning and disease duration are shown in Fig. 3.

**Fig. 4:**
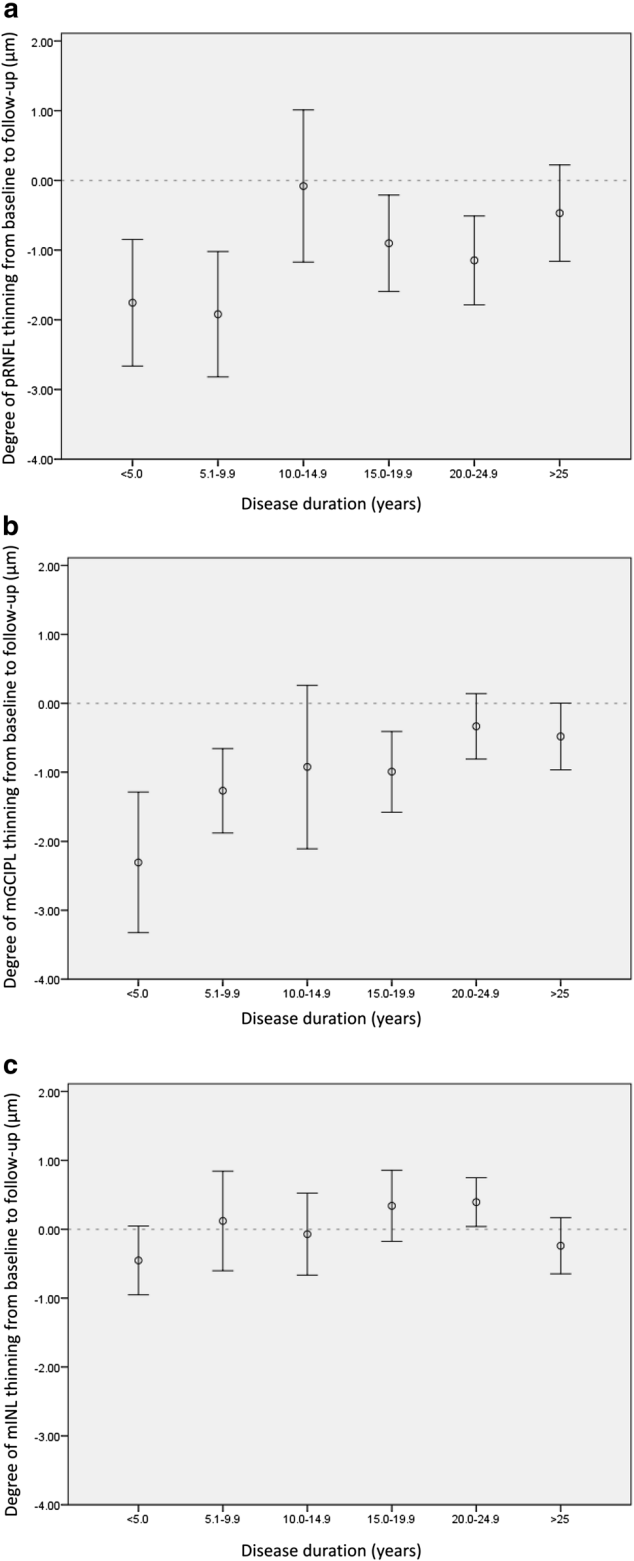
Mean change and 95 % CIs (error bars) in pRNFL (**a**), mGCIPL (**b**), and mINL (**c**) per disease duration group. pRNFL and mGCIPL thickness changes were dependent on disease duration, as changes were most pronounced early in the disease course. The horizontal dotted reference line indicates no change from baseline [group sizes: <5 years (*N* = 31), 5.0–9.9 years (*N* = 15), 10.0–14.9 years (*N* = 15), 15.0–19.9 years (*N* = 30), 20.0–24.9 (*N* = 17) and ≥25 years (*N* = 26)]

To investigate the association with disease duration in more detail, disease duration was categorized into six groups. Figure 4 shows the changes in retinal layer thickness for all disease duration categories. Thinning of the pRNFL and mGCIPL over the 2-year observation period was most pronounced in patients with a shorter disease duration. For the mINL, no such pattern was observed.

## Discussion

This study showed that over a 2-year observation period, significant thinning of the pRNFL and mGCIPL was observed throughout the course of the disease, but that these changes were significantly more pronounced early in the disease course. The attenuation of atrophy with longer disease duration is suggestive of a plateau effect. Our findings argue in favor of significant progressive neuronal and axonal injury occurring during the earliest stages of MS, providing support for early intervention to prevent or forestall this injury.

Establishing the timing of neuroaxonal loss in MS is crucial as we explore methods for assessing potential therapeutics meant to treat progressive disease and prevent this decline. It has been presumed in much of the literature that neuroaxonal loss is the substrate underlying volume loss on brain MRI. However, routinely used sequences on brain MRI lack specificity in this regard, making the retina an attractive additional site to monitor. Use of atrophy as a biomarker in progressive MS trials is dependent on the assumption that continued atrophy accumulates in the progressive phase of disease. This study helps to establish that the rate of atrophy in progressive disease may be lower and helps inform timing and sample size calculations for trials using OCT as an outcome.

In the present study, a longitudinal investigation of three retinal layers was performed, the pRNFL, mGCIPL and mINL. Firstly, our data showed significant thinning of the pRNFL (−1.1 µm), over 10 times more than observed in healthy reference subjects (−0.1 µm). This is in line with previously reported findings of longitudinal changes in pRNFL thickness obtained using time domain OCT [16, 18, 31]. In more recent studies using spectral-domain OCT, Narayanan and colleagues reported an annualized decrease in pRNFL of −1.49 μm for MSNON eyes, and −1.27 µm for MSON eyes, but they did not assess for the impact of disease duration or assess a cohort with a broad representation of disease durations [32]. Saidha and colleagues, however, reported a considerably smaller yearly decrease of −0.36 µm (MSON and MNON eyes together) [33].

Second, regarding the mGCIPL, we reported significant thinning over a 2-year observation period (−1.1 µm). This decrease in mGCIPL thickness, more than twice as much as observed in the reference population, was similar for MSON and MSNON eyes. In a recent longitudinal studies, Narayanan and colleagues reported comparable findings, with a yearly mGCIPL decrease of −0.53 µm in MSON and −0.49 in MSNON eyes [32], whereas Saidha et al. reported an annual change of −0.34 µm (MSON and MSNON together) [33].

Third, with respect to the mINL, the present study showed no consistent changes in patients with MS across the cohort, independent of disease duration or disease type. To our knowledge, no other longitudinal studies have investigated changes in mINL thickness. Interestingly, in human retinal postmortem samples, extensive loss of neurons in the mINL has been demonstrated after about 20 years of disease duration [20]. This discrepancy between in vivo and in vitro data would argue in favor of mINL thinning as being a dynamic process, instead of a constant process presenting a steady decrease during the disease course. This hypothesis is further strengthened by the reported increase in mINL thickness associated with MME. The occurrence of MME in MS [34], is characterized by the presence of cystic areas of hyporeflectivity in the mINL of the retina and it was shown that INL thickness changes were dynamic and were associated with increased disability [35]. Since none of the subjects experienced MME during the study, the present data do not allow for interpretation of the direct effect of MME on changes in mINL thickness, but we do not rule out the possibility of dynamic changes at a time scale not measured within the present study.

Taken together, available longitudinal literature consistently demonstrates progressive loss of pRNFL and mGCIPL in patients with MS. It was, however, previously unclear whether this inner retinal layer damage is a continuous process (as is often assumed with linear modeling), or if the underlying process is dependent on the stage of the disease. Previous studies have shown that retinal axonal injury was already present early in the disease course of MS [20–23]. Although the present data confirm this finding, we have also conclusively shown that the rate of pRNFL and mGCIPL thinning is dependent on disease duration and is greatest in early phases of the disease. Importantly, previous data on annual change of inner retinal layer thickness are inconsistent. Given the findings of the present study, we believe that annual change is clearly influenced by disease duration. Therefore, the inconsistent findings reported in literature are probably partly caused by unaccounted differences in disease duration between study cohorts.

Furthermore, whereas pRNFL and mGCIPL thinning were both present in eyes of patients with relapsing MS, the eyes of SPMS patients only showed substantial thinning of the mGCIPL. This finding is in concurrence with a previous longitudinal study by Henderson and colleagues, who reported no significant decrease in pRNFL thickness after an average follow-up of over 18 months in a cohort of progressive MS patients [19]. This finding suggests that although both neuronal and axonal injuries occur in the relapsing phase of the disease, only subtle neuronal damage continues in the progressive phase. Notwithstanding, this finding should be interpreted with caution, as the effect was [although statistically significant (*p* = 0.015)], only modestly stronger than observed in healthy references.

The present data support the large body of evidence on the effects of previous episode of MSON on pRNFL and mGCIPL thickness. Consistent with many other studies [10, 36–38], we demonstrated that the eyes of patients who experienced previous episodes of MSON showed significantly more damage of both inner layers at baseline, compared to unaffected (MSNON) eyes. The longitudinal data of the present study, however, extend to these findings, as we demonstrated that although the MSON eyes showed significantly more injury at baseline, the rate of thinning over time was similar for ON affected and unaffected eyes. Consequently, while prior episodes of MSON act as a major confounder in cross-sectional studies, its effect seems considerably less important in longitudinal investigations.

A major strength of the present study was the longitudinal design. Although cross-sectional studies have provided very useful and important data on retinal layer injury in MS, the longitudinal design of the present study, together with the very broadly representative cohort of patients, allowed for interpretation of the dynamics of retinal changes in different stages of MS. Although the 2-year observation period of the present study showed significant changes, the absolute changes were relatively small. Therefore, our current studies are focused on longer observation periods (3–5 years) with repeated OCT and clinical assessments. Our study may have several limitations. First, the control group was relatively small. Although this group was fairly homogeneous and similar to the patient population, a larger sample would increase statistical power. Similarly, although patients with a disease duration across the entire arc of disease were included, those with a progressive disease course (SPMS and PPMS) may have been underpowered, as the majority of patients had relapsing remitting MS. Second, extensive low-contrast visual acuity and color vision testing was not included in the present study. Both measures would provide important information on visual functioning and should be included in future studies [27].

In summary, this study showed that over a 2-year observation period, significant thinning of the pRNFL and mGCIPL, but not the mINL, was observed in patients with MS throughout the course of disease. These changes in pRNFL and mGCIPL were more pronounced early in the disease course. The attenuation of atrophy with longer disease duration suggests that the earliest phases of disease provide the optimal time to prevent permanent neuroaxonal injury.
